# Sex differences in higher-order cognition in schizophrenia and unaffected first-degree relatives: a longitudinal, family study

**DOI:** 10.1007/s00406-026-02292-0

**Published:** 2026-06-22

**Authors:** Vicent Balanzá-Martínez, Joan Vicent Sánchez-Ortí, Patricia Correa-Ghisays, Gabriel Selva-Vera, Tamsyn E. Van Rheenen, Rafael Tabarés-Seisdedos

**Affiliations:** 1 Teaching Unit of Psychiatry and Psychological Medicine, Department of Medicine, University of Valencia, INCLIVA, Blasco Ibáñez, 15, 46010 Valencia, Spain; 2 CIBERSAM, Instituto de Salud Carlos III, Madrid, Spain; 3 INCLIVA, Valencia, Spain; 4 Hospital Clínic Universitari, Valencia, Spain; 5 Melbourne Neuropsychiatry Centre, Department of Psychiatry, University of Melbourne and Melbourne Health, Melbourne, Australia; 6 Centre for Mental Health, School of Health Sciences, Swinburne University, Melbourne, Australia

**Keywords:** Executive function, Working memory, Schizophrenia, Sex differences, Family study

## Abstract

**Background:**

Although sex differences in the epidemiology and clinical expression of schizophrenia (SZ) are well established, sex differences in cognition during the chronic stage of SZ remains controversial and merits further investigation. We aimed to examine sex differences in executive function and working memory within a longitudinal family study of SZ, hypothesizing that sex effects would be negligible.

**Methods:**

A total of 266 participants, including 113 individuals with SZ, 58 unaffected first-degree relatives (SZR), and 95 healthy controls (HC), were assessed twice over one year using a comprehensive neuropsychological battery. Mixed one-way analyses of covariance were performed to compare these participant groups, accounting for sex.

**Results:**

No significant sex differences were found in performance on executive functioning and working memory tasks across SZ and SZR (*p* > 0.05). This performance pattern was also maintained for HC. However, the SZ group performed significantly worse than other two groups in most tasks (*p* < 0.0001; η²*p* = 0.09 to 0.14).

**Conclusion:**

The present results support previous evidence reporting the absence of sex differences in higher-order cognition in schizophrenia and provide new evidence that there are no sex effects in these domains in unaffected relatives of individuals with SZ.

**Supplementary Information:**

The online version contains supplementary material available at https://doi.org/10.1007/s00406-026-02292-0.

## Introduction

Neurocognitive impairment is a core feature of schizophrenia (SZ) and represents a major therapeutic target given its influence on functional outcomes [1–3]. Sex and gender differences in SZ have been reported in the context of its epidemiology, clinical expression, and course of illness [4–6]. Relatively reliable findings include a later age of onset in women compared to men; subtle differences in clinical symptoms, with men tending to have more negative, and women more affective symptoms; and better long-term course of illness, social functioning and recovery rates in women. However, the extent to which there are sex differences in neurocognition and social cognition in SZ remains controversial [7].

Although evidence does not provide strong support for sex differences on cognition in SZ [8], several reviews have revealed an inconsistent pattern of neurocognitive differences between men and women with SZ [4, 9, 10]. According to those reviews, some individual studies have reported worse neurocognitive functioning in men than in women, and others have reported an opposite pattern or no sex differences at all [4, 9, 10]. In cases in which sex differences have been found, the specific pattern of impairment across cognitive domains in men and women appears to differ from study to study [4]. Nonetheless, these studies generally tend to show that women perform better on processing speed and verbal memory tasks, whereas men perform better on tests of sustained attention [10, 11].

Discrepant findings across studies may result from differences in sample size, clinical characteristics, and cognitive domains assessed, and different neuropsychological tests used to assess the same cognitive domain, among other variables [4]. Research focused on specific stages of illness (e.g., first episode vs. chronic SZ) may help to clarify the extent to which there are sex differences in cognitive domains in SZ.

Studies of outpatients with SZ have found that men show worse global cognition than women [12], but better visuospatial/construction and language performance [13]. Other studies have found no sex differences in immediate memory, delayed memory, and attention [14, 15]. However, these studies did not examine higher-order cognition, such as working memory or executive functions, the latter of which is an umbrella domain comprising functions involved in planning, reasoning, abstraction, cognitive flexibility, and response inhibition [16]. Sex differences in higher-order cognition, particularly executive functioning, remain poorly investigated in both human and animal studies [10]. This is despite executive functions having been critical in our evolution as human beings [16] as well as a key determinant of functional outcomes in SZ [17, 18].

The sparse literature regarding sex differences in executive functions and working memory among individuals with SZ is not always consistent [7, 19]. One study [20] found that amongst stable outpatients, women with SZ performed lower on working memory. However, no sex differences were observed in cognitive flexibility and verbal abstract thinking [20]. Other studies found no differences between men and women in reasoning [11] and working memory [11, 21–23]. Moreover, age-related changes on most cognitive deficits, including working memory, showed no differences between men and women with SZ [24]. In other studies, SZ women had a greater impairment in reasoning/problem solving compared to SZ men [21, 23].

In healthy populations, research on sex differences is contradictory and inconclusive. Small but significant sex effects have been described in episodic memory, with men performing better than women in spatially demanding memory tasks, but worse in more verbal memory tasks [25]. Moreover, women outperform men in in recall and recognition verbal memory, as well as in phonemic fluency, but not in semantic fluency [26]. Meta-analytic evidence supports a female advantage in verbal working memory [27], and a small male advantage in spatial working memory, especially in mental rotation [28]. Regarding executive function tasks, a lack of sex differences was found in performance monitoring, response inhibition, and cognitive set-shifting [29]. Some evidence points to men showing increased impulsive action and reduced reaction time [30]. However, little support for significant sex differences in performance on executive control tasks was found in a recent review [30]. Collectively, cognitive sex differences among healthy individuals are generally small and are not as pronounced as commonly believed [8].

Unaffected relatives of individuals with SZ (SZR) also show deficits across the domains of verbal memory and psychomotor speed [31], sustained attention [32], and executive cognition (abstraction, cognitive flexibility, and response inhibition) [33]. Moreover, these deficits are milder in magnitude and SZR typically perform between SZ patients and healthy individuals [33, 34]. Even fewer studies have focused on sex differences in executive cognition and working memory among SZR. Moreover, the findings are mixed [35–37]. Relevantly, in the two existing *longitudinal* family studies of SZ (to our knowledge), the performance of SZR on executive tasks was similar for both men and women [38, 39].

Taken together, it is clear that sex differences in higher-order cognitive functioning (executive functions and working memory) are lacking systemic investigation in individuals with SZ and SZR. Indeed, to our knowledge, no previous study has characterized differences by sex in executive performance in a longitudinal, family study of SZ comprising three groups – patients, SZR, and healthy controls (HC). We aimed to bridge that gap within the present study.

Based on past evidence, we hypothesized that the effects of sex in performance on executive tasks and working memory would be negligible or insignificant across the three groups over a 1-year follow-up (Hypothesis 1). Moreover, patients with SZ were expected to show deficits in most cognitive functions, and SZR were expected to have performance that was intermediate to those with SZ and HC over a 1-year follow-up (Hypothesis 2).

## Materials and methods

### Study design and ethical considerations

This study is part of an ongoing larger neurocognitive investigation of severe mental disorders, carried out carried out between 2010 and 2015 by Group 24 CIBERSAM (Centro de Investigación Biomédica en Red de Salud Mental) / TMAP-UV (Unidad Autonomía Personal, Dependencia y Trastorno Mental Grave - Universitat de València) in Valencia, Spain. In this follow-up study, sociodemographic, clinical, and neurocognitive data were obtained simultaneously from individuals with SZ, their unaffected first-degree relatives (SZR), and genetically-unrelated healthy controls (HC). Healthy volunteers were residents of the same areas as those of individuals with psychiatric disorders. The investigation was carried out in accordance with the latest version of the Declaration of Helsinki. The investigation was carried out in accordance with the latest version Declaration of Helsinki [40, 41]. The Ethics Committee of the University Clinical Hospital of Valencia reviewed and approved the study, and all participants provided written informed consent after the nature of the procedures had been fully explained. In order to assess short-term neurocognitive trajectories, assessments were conducted at two timepoints (T1, T2), separated by 1–2 years after baseline (T1). For this study, only variables related to the present study aims were included in the analyses.

### Participants

This study assessed adult participants, including three groups – individuals with SZ (SZ) [men (SZ-M) and women (SZ-F)], SZ unaffected first-degree relatives (SZR) [men (SZR-M) and women (SZR-F)], and healthy controls (HC) [men (HC-M) and women (HC-F)]. The SZR group included biologically related parents, siblings, and offspring of the individuals in the SZ group. SZ was diagnosed according to the criteria of the Diagnostic and Statistical Manual of Mental Disorders; DSM IV-TR [42] or DSM-5 [43]. Participants in the SZ group were all in chronic stages, defined as at least 5 years post illness onset. The following inclusion criteria had to be fulfilled. For individuals with SZ: (1) clinical stability was required, as defined by the criteria of Andreasen et al. (2005) [44]. Specifically, participants were required to be clinically stable outpatients, with no psychiatric hospitalization or acute exacerbation of psychotic symptoms in the previous 3–6 months and no significant changes in antipsychotic medication type or dosage during the last 4–8 weeks. Clinical stability was confirmed through clinical interview and review of medical records [44–46], (2) had not participated structured cognitive remediation or cognitive enhancement programs within the 6 months prior to study entry, and (3) had not received electroconvulsive therapy (ECT) within the last 12 months. For SZR: (1) no personal history or current diagnosis of severe mental disorder, defined as psychotic, bipolar disorder and major depressive disorder, and (2) currently not taking psychotropic medication. For HC: 1) absence of family and personal history of psychotic and mood disorders as ascertained by expert psychiatrists through a personal clinical interview. The exclusion criteria for all participants in this study were: (1) age lower than 18 years (and higher than 65 years for SZ and HC groups), (2) presence of any neurological disorder, history of brain injury or any medical condition that could interfere with neurocognitive performance, (3) intellectual disability (IQ < 70), as estimated with the Vocabulary subtest from the Wechsler Adult Intelligence Scale (WAIS III) [47, 48], and (4) current substance use disorder, except for nicotine, or being under the influence of any substance at the time of assessment.

### Clinical and neuropsychological assessments

Assessments were conducted by the same experienced psychologists and psychiatrists of the research group (G24 CIBERSAM/TMAP-UV). Sociodemographic data, including sex (defined as biological condition), age, years of education, occupational status, and hand dominance, were collected. Clinical variables, such as age at onset, duration of illness, and symptom severity, were collected for descriptive purposes. The following instruments were used: (i) 17-item Hamilton Rating Scale for Depression (HDRS) [49], (ii) Young Mania Rating Scale [50], (iii) Positive and Negative Syndrome Scale (PANSS) [51]. Herein, we align with Kheloui et al. (2023) in that the term ‘sex/gender differences’ represents the combination and interrelation of sex and gender factors in cognitive functioning [52].

Neurocognitive performance was evaluated using a comprehensive battery of neuropsychological tests previously used by our group [53–64]. For the present study, four higher-order tests were used to assess the following cognitive functions: (i) *abstract reasoning*: Wisconsin Card Sorting Test (WCST) categories completed [65]; (ii) *cognitive flexibility*: WCST perseverative errors [65]; (iii) *response inhibition*: Stroop Color and Word test (SCWT) color/word subtest [66]; and (iv) *working memory*: WAIS-III digit span backwards [48]. In addition, premorbid intelligence quotient (IQ) was estimated using the WAIS-III Vocabulary subtest.

### Statistical analyses

Data were analyzed using Statistical Package for Social Sciences version 26.0 for Windows [67]. Preliminary descriptive analyses comparing groups in age, years of education, and hand dominance were conducted using a one-way analysis of variance (ANOVA) for continuous variables and the Chi-square test for categorical variables. The raw scores obtained for each neurocognitive test were transformed into Z-scores using the means and standard deviations of the HC group at T1 as reference values. Scores quantifying the number of errors (i.e., WCST perseverative errors) were multiplied by − 1 so that lower Z-scores consistently indicated poorer performance.

To address Hypothesis 1, we first split each diagnostic group by sex and compared their neurocognitive performance at T1 and T2 and their evolution over time using mixed one-way analysis of covariance (MANCOVA). Variables where significant group effects were identified were entered as covariates.

To address Hypothesis 2, we examined differences between each diagnostic group in cognitive functions at T1 and T2 and their evolution over time using mixed MANCOVA. Variables where significant group effects were identified were entered as covariates. Sex was not included as a covariate due to its examination in the analysis testing Hypothesis 1.

Normality was assumed for all continuous variables because the sample was statistically verified using Kolmogorov-Smirnov test. *Post hoc* analyses with Bonferroni-corrected pairwise t-tests and the Mann−Whitney U tests were performed to examine differences between these groups. Effect sizes were calculated with partial eta-squared (η^²^p), and the following values were used as reference: small = 0.02, moderate = 0.15, and large = 0.35. For all analyses, *p* < 0.05 indicated statistical significance.

## Results

### Preliminary analysis

The overall sample at T1 was composed of 352 participants in total, including 150 SZ, 85 SZR, and 117 HC. Only participants who completed both neurocognitive assessments (T1 and T2) were considered in the present analysis. Thus, the sample consisted of 266 individuals (48% women) at both timepoints, including 81 SZ-M, 32 SZ-F, 22 SZR-M, 36 SZR-F, 36 HC-M, and 59 HC-F. A summary of the baseline sociodemographic characteristics of participants in each sex-segregated group is presented in Table 1. The mean age of the total sample was 41.2 (SD: 13.1) years. The HC groups were characterized by having significantly higher years of education than the other groups. The SZR groups were significantly older than the other groups. Hand dominance was similar across all groups. The baseline sociodemographic characteristics of the three full groups, irrespective of sex, are provided in Supplementary Table 1. The clinical characteristics of the schizophrenia group are presented in Supplementary Table 1. Significant differences were found in age, sex, and years of education. The sex distribution did not differ significantly between diagnostic groups. Age and years of education were included as covariates in the statistical models when appropriate.

**Table 1:** Sociodemographic characteristics of the sample at Time 1

Variables^a^	HC-M	HC-F	SZ-M	SZ-F	SZR-M	SZR-F	Statistical analyses	
	(*n* = 36)	*(n* = 59)	(*n* = 81)	(*n* = 32)	(*n* = 22)	(*n* = 36)	F(*p*)^c^	Post hoc test^d^
Age	36.7(13.0)	36.0(12.6)	36.2(8.8)	38.8(10.0)	50.8(17.7)	49.1(16.5)	10.2****	HC-M, HC-F, SZ-M, SZ-F < SZR.M, SZR-F
Years of education	14.2(4.1)	13.5(3.5)	10.3(3.1)	10.9(3.9)	8.5(4.1)	7.9(4.0)	18.3****	SZR-M, SZR-F, SZ-M, SZ-F < HC-M, HC-F SZR-F < SZ-M, SZ-F
Hand dominance^**b**^	32(89%)	51(86%)	75(93%)	29(91%)	21(95%)	33(92%)	NS	
IQ	119.2(12.0)	114.9(15.2)	96.9(17.7)	97.1(14.3)	96.8(17.9)	94.0(12.2)	11.7****	SZR-M, SZR-F, SZ-M, SZ-F < HC-M, HC-F

*T1* time 1,  *M* men, *F * women, *HC* healthy control, *SZ* schizophrenia, *SZR* schizophrenia relative, *IQ* intellectual quotient, *ANOVA* analysis of variance, *NS* not significant

^a^ Expressed as mean(standard deviation) except when indicated

^b^ right-handers n (%)

^c^ ANOVA

^d^ Bonferroni test

(NS = *p* > 0.05; *p ≤ 0.05; **p ≤ 0.01; ***p ≤ 0.001; ****p ≤ 0.0001)

### Sex differences in neurocognitive performance (hypothesis 1)

The neurocognitive performance of the six sex-segregated SZ, SZR and HC groups at T1 and T2 is presented in Table 2. At T1, abstract reasoning was significantly lower in SZ-M compared to both HC groups (*p* < 0.0001; η²*p* = 0.09). Likewise, cognitive flexibility and working memory were significantly lower in SZ-M compared to SZR groups and HC groups (*p* < 0.0001; η²*p* = 0.13). At T2, significant differences in abstract reasoning, cognitive flexibility and working memory were expanded to SZ-F (*p* < 0.0001; η²*p* = 0.14). Moreover, SZ-F exhibited significantly lower cognitive flexibility than SZR-M and HC groups at T2 (*p* < 0.0001; η²*p* = 0.15). Finally, response inhibition was similar across all six groups (*p* > 0.05). Small-to-moderate effect sizes were observed for cognitive functions in between-group comparisons at both assessments. No significant within-group differences were observed in neurocognitive performance over time (*p* < 0.05 in all cases). Notably, no significant within-group sex differences in executive functioning were observed in any of the three groups (SZ, SZR, HC) (*p* < 0.05), either at baseline or at 1-year follow-up. Raw neurocognitive performances (means and standard deviations) of sex-segregated groups are provided in Supplementary Table 2.

**Table 2:** Neurocognitive performance. Between-group comparison at Time 1 and Time 2 (Z-scores)

Variables^a^	HC-M (*n* = 36)		HC-F *(n* = 59)		SZ-M (*n* = 81)		SZ-F (*n* = 32)		SZR-M (*n* = 22)		SZR-F (*n* = 36)		Statistical analyses					
	T1	T2	T1	T2	T1	T2	T1	T2	T1	T2	T1	T2	T1 F(*p*)^b^	Post hoc test^c^	η²*p*^d^	T2 F(*p*)^b^	Post hoc test^c^	η²*p*^d^
WCST categories	0.1(0.8)	0.3(0.6)	-0.1(1.1)	0.2(0.8)	-1.0(0.8)	-0.8(1.0)	-0.8(0.9)	-0.6(0.9)	-0.9(1.0)	-0.7(1.0)	-1.1(0.9)	-0.9(1.0)	5.5****	SZ-M, SZ-F < HC-M SZ-M < HC-F	0.09	8.5****	SZ-M, SZ-F < HC-M, HC-F	0.14
WCST perseverative errors	0.2(0.6)	0.3(0.8)	-0.1(1.1)	0.1(1.0)	-1.7(2.9)	-1.8(2.9)	-1.5(2.7)	-1.5(2.3)	-0.6(1.5)	-0.3(1.8)	-0.9(1.2)	-0.7(1.4)	7.6****	SZ-M < SZR-M, SZR-F, HC.M, HC-F SZ-F < HC-M	0.13	9.2****	SZ-M < SZR-M, SZR-F, HC-M, HC-F SZ-F < SZR-M, HC-M, HC-F	0.15
SCWT Colour-Word	0.1(0.8)	0.1(0.9)	-0.1(1.0)	-0.2(1.2)	-0.1(0.9)	-0.2(0.9)	-0.2(1.1)	-0.3(1.0)	-0.1(0.5)	-0.1(0.6)	-0.2(0.7)	-0.2(0.7)	NS			NS		
WAIS-III digit span backwards	0.1(1.1)	0.3(1.1)	-0.1(0.8)	-0.1(0.9)	-1.0(0.7)	-0.9(0.8)	-1.0(0.8)	-1.0(0.7)	-0.6(0.9)	-0.7(0.8)	-0.9(0.7)	-1.0(0.8)	9.3****	SZ-M, SZ-F < HC-M, HC-F SZ-M, SZ-F < SZR-M	0.15	9.5****	SZ-M, SZ-F < HC-M, HC-F SZR-F < HC-M	0.15

*T1* time 1, *T2* time 2, *M * men, *F* women, *HC* healthy control, *SZ* schizophrenia, *SZR* schizophrenia relative, *WCST* Wisconsin Card Sorting Test, *SCWT* Stroop Color and Word test, *WAIS-III* Wechsler Adult Intelligence Scale III edition, *MANCOVA* analysis of co-variance, *NS* not significant

^a^ Expressed as mean(standard deviation)

^b^ MANCOVA

^c^ Bonferroni test

^d^ Partial Eta-Squared (η²p)

(NS = *p* > 0.05; *p ≤ 0.05; **p ≤ 0.01; ***p ≤ 0.001; ****p ≤ 0.0001). Effect size (η²p: small ≈ 0.02; moderate ≈ 0.15; large ≈ 0.35)

### Between-group comparisons of neurocognitive performance (hypothesis 2)

The comparison of neurocognitive performance across the three full (non sex-segregated) diagnostic groups (SZ, SZR, HC) at T1 and T2 appears in Table 3. Compared to HCs, the full SZ group performed worse on abstract reasoning, cognitive flexibility and working memory at T1 and T2 (*p* < 0.0001; η²*p* = 0.08–0.14). Moreover, the full SZ group showed worse performance than the SZR group on cognitive flexibility and working memory at T1, and on abstract reasoning, cognitive flexibility and working memory at T2. Likewise, the full SZR group underperformed HC in abstract reasoning as well as in working memory, but only at T2 (*p* < 0.0001; η²*p* = 0.13–0.14). Raw neurocognitive performances (means and standard deviations) of the three full diagnostic groups are provided in Supplementary Table 3.

**Table 3:** Neurocognitive performance by diagnosis. Between-group comparison at Time 1 and Time 2 (Z-scores)

Variables^a^	HC (*n* = 95)		SZ (*n* = 113)		SZR (*n* = 58)		Statistical analyses					
	T1	T2	T1	T2	T1	T2	T1 F(*p*)^b^	Post hoc test^c^	η²*p*^d^	T2 F(*p*)^b^	Post hoc test^c^	η²*p*^d^
WCST Categories	0.0(1.0)	0.3(0.8)	-0.9(0.9)	-0.8(1.0)	-1.1(1.0)	-0.8(1.0)	12.1****	SZ < HC	0.08	20.6****	SZ, SZR < HC SZ < SZR	0.13
WCST Perseverative errors	0.0(1.0)	0.1(0.9)	-1.6(2.6)	-1.7(2.7)	-0.8(1.3)	-0.4(1.2)	18.2****	SZ < SZR, HC	0.12	21.8****	SZ < SZR, HC	0.14
SCWT Colour-Word	0.0(1.0)	0.1(1.1)	-0.1(0.9)	-0.2(0.9)	-0.1(0.7)	-0.1(0.7)	NS			NS		
WAIS-III Digit span backwards	0.0(1.0)	0.1(1.0)	-1.0(0.7)	-0.9(0.7)	-0.8(0.8)	-0.9(0.8)	21.9****	SZ < SZR, HC	0.14	22.0****	SZ, SZR < HC SZ < SZR	0.14

*T1* time 1, *T2* time 2, *HC * healthy control, *SZ* schizophrenia, *SZR* schizophrenia relative, *WCST* Wisconsin Card Sorting Test, *SCWT* Stroop Color and Word test, *WAIS-III* Wechsler Adult Intelligence Scale III edition, *MANCOVA* analysis of co-variance, *NS* not significant

^a^ Expressed as mean(standard deviation)

^b^ MANCOVA

^c^ Bonferroni test

^d^ Partial Eta-Squared (η²p)

(NS = *p* > 0.05; *p ≤ 0.05; **p ≤ 0.01; ***p ≤ 0.001; ****p ≤ 0.0001). Effect size (η²p: small ≈ 0.02; moderate ≈ 0.15; large ≈ 0.35)

## Discussion

The major finding of this longitudinal family study is that performance on four higher-order tests was independent of sex for the three groups. That is, in keeping with Hypothesis 1, no significant sex differences were found in performance on executive functioning and working memory tasks across individuals with SZ and SZR. This pattern was also maintained for healthy controls.

The lack of sex differences in cognition performance in all groups in the present study is not surprising given the previous literature is quite mixed. Indeed, the pattern of sex effects in the SZ literature is inconsistent and a matter of much discussion [see reviews: 4, 7, 9, 10]. Overall, previous evidence does not provide strong support for differential sex effects on executive cognition [20, 68] and working memory [11, 21–24, 68, 69] in SZ.

Much less research has been conducted in unaffected relatives of individuals with SZ. Findings regarding potential sex differences on higher-order cognition are mixed in SZR [35, 36]. Longitudinal, family studies support a similar performance on abstract reasoning and cognitive flexibility for both men and women SZR [38, 39]. Moreover, the lack of sex effects in executive cognition and working memory among healthy individuals also concur with recent meta-analytic evidence [27–30].

Overall, we agree with the conclusions of a recent review [8] that despite it is possible that SZ may be associated with sex differences in certain cognitive domains, that effect might not be strong enough to be consistently found across studies, especially in those with a small sample size.

Regarding our second hypothesis, individuals with SZ showed persistent deficits across abstract reasoning, cognitive flexibility and working memory. These results are consistent with prior research [70, 71]. In keeping with neurocognitive endophenotypes, unaffected relatives’ pattern of performance was intermediate between the other groups [33, 34, 72]. Moreover, unaffected relatives showed deficits in abstract reasoning and working memory although those deficits were not stable over time [32, 73].

Further research is warranted. It has been suggested that the characterization of potential sex differences in neurocognition of individuals with SZ may advance our understanding on the pathogenetic mechanisms subserving neurocognitive impairment in SZ and on their functional impact [7]. In addition, more family studies are needed. Research on unaffected biological relatives may identify sex-specific neurocognitive impairments associated with the illness while avoiding the effects of psychopathology and medications, and ultimately develop sex-tailored rehabilitation strategies [21].

Strengths of the study include the family study design, as few existing family studies of SZ have included a comprehensive characterization of executive cognition in SZ. Moreover, the longitudinal nature of the study allowed for repeated observation of the associations between sex and cognition in the same SZ and unaffected relatives over time, which has not been of research focus so far. Lastly, the study focused explicitly on higher-order cognitive domains that have been shown to be key for individuals’ daily life performance [16] and represent major therapeutic targets for cognitive-enhancing strategies in SZ [74, 75]. These domains have not been of significant research focus in the context of sex-differences in SZ to date.

Despite these strengths, the study has several limitations. Like many similar studies examining sex differences in cognition in SZ, we did not consider the different phases of the menstrual cycle or sex hormones, e.g., oestrogen levels, which may account for some neurocognitive differences by sex [76, 77]. In addition, the study groups were not matched in variables that may affect neurocognition. Indeed, the SZR group was, on average, older and less educated than the SZ and HC groups. However, both variables were statistically controlled in the analyses. Although the schizophrenia group included a lower proportion of women compared to other groups, analyses stratified by sex revealed no significant differences between sexes in cognitive functions, indicating that this imbalance did not influence the study results. Standardized instruments (e.g., SCID or CIDI) were not used to assess family and personal psychiatric history of healthy controls. Finally, we analysed a relatively small sample of participants, which was related in part to the requirement for repeated assessments. Nonetheless, the present sample size can be considered relatively standard for longitudinal family studies of SZ [38, 39].

In conclusion, no sex differences in higher order cognition were found across any of the participant groups of interest in this study. The present results support previous evidence reporting the absence of sex differences in executive cognition and working memory in SZ. Moreover, we provide new evidence, based on a longitudinal, family study, that there are no sex effects in these domains in unaffected relatives of individuals with SZ. Together, sex differences in cognition in SZ and their unaffected relatives do not exist or are likely to be negligible.

## Supplementary Information

Below is the link to the electronic supplementary material.


Supplementary Material 1


## Data Availability

The data that support the findings of this study are available from the corresponding author upon reasonable request.
