# Supplementary material for: Sex differences in higher-order cognition in schizophrenia and unaffected first-degree relatives: a longitudinal, family study
Source: Eur Arch Psychiatry Clin Neurosci. 2026 Jun 22;276(6):3173–81. doi: 10.1007/s00406-026-02292-0 (PMC13526111; doi:10.1007/s00406-026-02292-0)
Supplement: Supplementary file 1 [file 406_2026_2292_MOESM1_ESM.docx]

| **Supplementary Table 1.** **Sociodemographic characteristics of the sample by diagnostic group at T1** | | | | | |
| --- | --- | --- | --- | --- | --- |
|  | **HC** | **SZ** | **SZR** | ***Statistical analyses*** | |
| **Variables^a^** | (*n*=95) | (*n*=113) | (*n*=58) | **F(*p*)^f^** | **Post hoc test^h^** |
| *Sociodemographic* | | | | | |
| **Sex^b,g,i^** | 60(63%) | 32(28%) | 36(62%) | 30.8**** | SZ<SZR,HC |
| **Age** | 36.3(12.7) | 36.9(9.2) | 49.7(16.8) | 25.1**** | HC,SZ<SZR |
| **Years of education** | 13.8(3.8) | 10.4(3.3) | 8.1(4.0) | 45.2**** | SZR,SZ<HC  SZR<SZ |
| **Hand dominance^c^** | 83(87%) | 104(92%) | 54(93%) | NS |  |
| **IQ** | 116.2(14.3) | 98.4(17.1) | 99.4(15.8) | 25.5**** | SZ,SZR<HC |
| *Clinical* | | | | | |
| **Age of onset^d^** |  | 25.6(8.0) |  |  |  |
| **Illness duration^d^** |  | 15.2(8.4) |  |  |  |
| **HRSD^e^** |  | 7.0(5.8) |  |  |  |
| **YMRS^e^** |  | 3.2(4.9) |  |  |  |
| **PANSS-T^e^** |  | 20.3(9.0) |  |  |  |
| ^a^ Expressed as mean (standard deviation) except when indicated, ^b^ female n (%), ^c^ right-handers n (%),^d^ years, ^e^ lower scores represent a better outcome. ^f^ ANOVA. ^g^ chi-square test; ^h^ Bonferroni test; ^i^ Mann–Whitney U test. Abbreviations: T1 = time 1, HC = healthy control, SZ = schizophrenia, SZR = schizophrenia relative, IQ = intellectual quotient, HRSD = Hamilton rating scale for depression, YMRS = Young mania rating scale, PANSS = positive and negative syndrome scale, T = total, ANOVA = analysis of variance, NS = not significant. (NS = p > 0.05; *p ≤ 0.05; **p ≤ 0.01; ***p ≤ 0.001; ****p ≤ 0.0001). | | | | | |

| **Supplementary Table 2. Raw neurocognitive performance of sex-segregated groups** | | | | | | | | | | | | |
| --- | --- | --- | --- | --- | --- | --- | --- | --- | --- | --- | --- | --- |
|  | **HC-M** (*n*=36) | | **HC-F** *(n*=59) | | **SZ-M** (*n*=81) | | **SZ-F** (*n*=32) | | **SZR-M** (*n*=22) | | **SZR-F** (*n*=36) | |
| **Variables^a^** | **T1** | **T2** | **T1** | **T2** | **T1** | **T2** | **T1** | **T2** | **T1** | **T2** | **T1** | **T2** |
| **WCST Categories** | 4.3(1.2) | 4.7(1.0) | 3.9(1.6) | 4.5(1.3) | 2.5(1.3) | 2.7(1.6) | 2.8(1.4) | 3.0(1.5) | 2.6(1.6) | 2.9(1.6) | 2.3(1.4) | 2.6(1.5) |
| **WCST Perseverative errors** | -6.9(3.2) | -6.8(4.4) | -9.3(5.9) | -7.9(5.6) | -17.7(15.4) | -18.0(15.3) | -16.5(9.0) | -16.5(12.1) | -11.8(8.1) | -10.6(4.2) | -13.3(6.7) | -12.5(7.4) |
| **SCWT Colour-Word** | -22.8(10.6) | -23.3(11.9) | -26.2(12.9) | -28.6(15.3) | -25.9(11.0) | -27.6(11.6) | -28.3(13.8) | -29.4(12.2) | -25.5(7.1) | -26.3(8.2) | -28.2(9.4) | -28.0(9.2) |
| **WAIS-III Digit span backwards** | 5.3(1.6) | 5.6(1.6) | 5.1(1.2) | 5.1(1.3) | 3.7(1.1) | 3.8(1.1) | 3.7(1.1) | 3.7(0.9) | 4.2(1.3) | 4.1(1.2) | 3.8(1.1) | 3.7(1.2) |
| ^a^ Expressed as mean (standard deviation). Abbreviations: T1 = time 1, T2 = time 2, M= men, F= women, HC = healthy control, SZ = schizophrenia, SZR = schizophrenia relative, WCST: Wisconsin Card Sorting Test, SCWT: Stroop Color and Word test, WAIS-III: Wechsler Adult Intelligence Scale III edition. Negative values reflect the calculation of the Stroop interference score, in which lower scores indicate poorer performance in response inhibition. | | | | | | | | | | | | |

| **Supplementary Table 3. Raw neurocognitive performance score by diagnostic group** | | | | | | |
| --- | --- | --- | --- | --- | --- | --- |
|  | **HC** (*n*=95) | | **SZ** (*n*=113) | | **SZR** (*n*=58) | |
| **Variables^a^** | **T1** | **T2** | **T1** | **T2** | **T1** | **T2** |
| **WCST Categories** | 4.1(1.5) | 4.6(1.2) | 2.6(1.3) | 2.8(1.5) | 2.4(1.5) | 2.7(1.5) |
| **WCST Perseverative errors** | -8.4(5.2) | -7.5(5.2) | -17.3(13.9) | -17.5(14.4) | -12.7(7.2) | -11.0(6.6) |
| **SCWT Colour-Word** | -25.0(12.1) | -26.6(14.2) | -26.6(11.8) | -28.1(11.8) | -27.2(8.7) | -27.3(8.8) |
| **WAIS-III Digit span backwards** | 5.2(1.4) | 5.3(1.4) | 3.7(1.1) | 3.8(1.0) | 3.9(1.2) | 3.9(1.2) |
| ^a^ Expressed as mean (standard deviation). Abbreviations: T1 = time 1, T2 = time 2, HC = healthy control, SZ = schizophrenia, SZR = schizophrenia relative, WCST: Wisconsin Card Sorting Test, SCWT: Stroop Color and Word test, WAIS-III: Wechsler Adult Intelligence Scale III edition. Negative values reflect the calculation of the Stroop interference score, in which lower scores indicate poorer performance in response inhibition. | | | | | | |
